# Progestin primed ovarian stimulation using corifollitropin alfa in PCOS women effectively prevents LH surge and reduces injection burden compared to GnRH antagonist protocol

**DOI:** 10.1038/s41598-021-02227-w

**Published:** 2021-11-23

**Authors:** Ting-Chi Huang, Mei-Zen Huang, Kok-Min Seow, Ih-Jane Yang, Song-Po Pan, Mei-Jou Chen, Jiann-Loung Hwang, Shee-Uan Chen

**Affiliations:** 1 Department of Obstetrics and Gynecology, National Taiwan University Hospital, No. 7 Chung-Shan South Road, Taipei, Taiwan; 2 Department of Nursing, National Tainan Junior College of Nursing, Tainan, Taiwan; 3 Department of Obstetrics and Gynecology, School of Medicine, National Yang Ming Chiao Tung University, Taipei, Taiwan; 4 Department of Obstetrics and Gynecology, Shin Kong Wu Ho-Su Memorial Hospital, Taipei, Taiwan; 5 Taipei IVF, Center for Reproduction and Genetics, Taipei, Taiwan; 6 Department of Obstetrics and Gynecology, Taipei Medical University, Taipei, Taiwan

**Keywords:** Endocrine reproductive disorders, Infertility

## Abstract

Utilizing corifollitropin alfa in GnRH antagonist (GnRHant) protocol in conjunction with GnRH agonist trigger/freeze-all strategy (corifollitropin alfa/GnRHant protocol) was reported to have satisfactory outcomes in women with polycystic ovary syndrome (PCOS). Although lessening in gonadotropin injections, GnRHant were still needed. In addition to using corifollitropin alfa, GnRHant was replaced with an oral progestin as in progestin primed ovarian stimulation (PPOS) to further reduce the injection burden in this study. We try to investigate whether this regimen (corifollitropin alfa/PPOS protocol) could effectively reduce GnRHant injections and prevent premature LH surge in PCOS patients undergoing IVF/ICSI cycles. This is a retrospective cohort study recruiting 333 women with PCOS, with body weight between 50 and 70 kg, undergoing first IVF/ICSI cycle between August 2015 and July 2018. We used corifollitropin alfa/GnRHant protocol prior to Jan 2017 (n = 160), then changed to corifollitropin alfa/PPOS protocol (n = 173). All patients received corifollitropin alfa 100 μg on menstruation day 2/3 (S1). Additional rFSH was administered daily from S8. In corifollitropin alfa/GnRHant group, cetrorelix 0.25 mg/day was administered from S5 till the trigger day. In corifollitropin alfa/PPOS group, dydrogesterone 20 mg/day was given from S1 till the trigger day. GnRH agonist was used to trigger maturation of oocyte. All good quality day 5/6 embryos were frozen, and frozen-thawed embryo transfer (FET) was performed on subsequent cycle. A comparison of clinical outcomes was made between the two protocols. The primary endpoint was the incidence of premature LH surge and none of the patients occurred. Dydrogesterone successfully replace GnRHant to block LH surge while an average of 6.8 days of GnRHant injections were needed in the corifollitropin alfa/GnRHant group. No patients suffered from ovarian hyperstimulation syndrome (OHSS). The other clinical outcomes including additional duration/dose of daily gonadotropin administration, number of oocytes retrieved, and fertilization rate were similar between the two groups. The implantation rate, clinical pregnancy rate, and live birth rate in the first FET cycle were also similar between the two groups. In women with PCOS undergoing IVF/ICSI treatment, corifollitropin alfa/PPOS protocol could minimize the injections burden with comparable outcomes to corifollitropin alfa/GnRHant protocol.

## Introduction

Polycystic ovary syndrome (PCOS) affects 5–10% of reproductive-age women and accounts for approximately 80% of cases of anovulatory infertility^1^. After several cycles of failed ovulation induction, in vitro fertilization (IVF)/intracytoplasmic sperm injection (ICSI) is an effective treatment to achieve pregnancy in infertile women with PCOS^2^. Women with PCOS are prone to develop ovarian hyperstimulation syndrome (OHSS) during IVF/ICSI treatment^2,3^, which can be prevented by a gonadotropin releasing hormone agonist (GnRHa) to trigger final oocyte maturation in a GnRH antagonist (GnRHant) protocol^4^. However, GnRHa trigger results in lower pregnancy and implantation rates due to a defective luteal phase in the fresh embryo transfer cycle. This can be avoided by luteal phase support with intensive estrogen/progesterone or low-dose human chorionic gonadotropin (hCG) if fresh embryo transfer is intended^5^. The other strategy is to electively freeze all embryos with subsequent frozen-thawed embryo transfer (FET), which substantially eliminates OHSS^4^. In a prospective randomized controlled trial (RCT) that recruited 1508 infertile women with PCOS undergoing first IVF/ICSI cycle in a GnRHant protocol, an elective freeze-all strategy with subsequent FET resulted in a statistically significantly higher live birth rate and lower risk for OHSS compared with fresh embryo transfer^6^. Taken together, a GnRHant cycle in combination with the GnRHa trigger and freeze-all (GnRHa trigger/freeze-all) strategy has been advocated to be an ideal protocol for woman with PCOS women undergoing IVF/ICSI treatment in terms of safety and clinical outcomes^7–9^.

Corifollitropin alfa is a long-acting follicle stimulating hormone (FSH) designed to reduce the injection burden on patients during controlled ovarian stimulation (COS). A single dose of corifollitropin alfa can induce and maintain follicular growth for 7 days^10^. A RCT and a recent systematic review and meta-analysis reported that the use of corifollitropin alfa in GnRH antagonist protocols produced similar live birth and ongoing pregnancy rates similar to daily recombinant FSH (rFSH) administration in normal and poor responder patients undergoing IVF/ICSI treatment cycles^11,12^.

Corifollitropin alfa has seldom been studied in PCOS patients due to concerns for excessive ovarian stimulation and OHSS. To reduce the injection burden of daily gonadotropin administration and minimize the risk for OHSS in PCOS patients undergoing IVF/ICSI treatment, we used corifollitropin alfa in a GnRHant protocol by combing the GnRHa trigger/freeze-all strategy (corifollitropin alfa/GnRHant protocol)^13^. A single dose of corifollitropin alfa was used for the initial 7 days of COS. No patients experienced OHSS and satisfactory clinical outcomes were achieved. It was a patient friendly protocol compared to those involving daily gonadotropin injections. However, GnRHant injections were still needed to prevent premature luteinizing hormone (LH) surge^13^.

Kuang et al. recently proposed progestin primed ovarian stimulation (PPOS) protocol by using oral progestin in the follicular phase during COS as an effective alternative to GnRHa and GnRHant to prevent LH surge in women undergoing IVF/ICSI treatment^14^. Daily administration of medroxyprogesterone acetate (MPA), micronized progesterone (MIP), or dydrogesterone during COS has been reported to effectively block LH surge during COS^15^. In this study we attempted to replace GnRHant with dydrogesterone to prevent premature LH surge in PCOS women undergoing IVF/ICSI treatment to further reduce the injection burden of GnRHant. As in corifollitropin alfa/GnRHant protocol^13^, corifollitropin alfa was used for the first 7 days of COS and GnRHa trigger/freeze-all strategy was applied to minimize the risk for OHSS. In this study, this protocol (corifollitropin alfa/PPOS protocol) was applied in women with PCOS, weighing between 50 and 70 kg who were undergoing their first IVF/ICSI treatment. We hypothesized that this strategy would effectively prevent a premature LH surge and reduce the injection burden of GnRHant. The primary endpoint of the study was the incidence of premature LH surge. We also attempted to compare the clinical outcomes of corifollitropin alfa/PPOS and corifollitropin alfa/GnRHant protocols.

## Materials and methods

### Study design

The present investigation was a retrospective cohort study. A review of medical records of PCOS patients, weighing between 50 and 70 kg, undergoing their first IVF/ICSI treatment at National Taiwan University Hospital and Taipei IVF between August 2015 and July 2018 was performed. A total of 6684 IVF/ICSI cycles were performed during the study period. There were 875 PCOS patients, 475 of them were of their first IVF/ICSI cycle. Finally, 333 PCOS patients were included for analysis (Supplementary Data). Institutional Review Board approval was obtained from the National Taiwan University Hospital (201811078RINB). In PCOS patients undergoing IVF/ICSI treatment, the GnRHa trigger/freeze-all strategy is routinely used in the units to minimize the risk for OHSS. Before to January 2017, GnRHant was used to prevent premature LH surge. Subsequently, GnRHant was replaced with dydrogesterone. Regarding gonadotropins selection at the first treatment cycle, corifollitropin alfa at a dose of 100 μg (Elonva; NV Organon, Oss, The Netherlands) was used for the first 7 days of ovarian stimulation if the patient’s body weight was between 50 and 70 kg. Otherwise, daily FSH was the drug of choice for COS. Daily rFSH (Gonal-f; Merck Serono, Modungo, Italy) 200 IU was administered for the first 4 days of COS in patients with a body weight > 70 kg. For patients with a body weight < 50 kg, rFSH 112.5 IU per day was administered.

### Participants

The present study recruited infertile women with PCOS, with a body weight between 50 and 70 kg, undergoing their first IVF/ICSI cycle. PCOS was diagnosed according to the Rotterdam consensus criteria (two out of three of the following criteria: oligo- or anovulation, clinical and/or biochemical signs of hyperandrogenism and polycystic ovaries)^16^. A diagnosis of congenital adrenal hyperplasia, Cushing's syndrome, androgen-producing tumours, hyperprolactinemia and thyroid dysfunction were all rulled out. Exclusion criteria were as follows: age > 38 years, basal FSH level > 12 mIU/mL, previous ovarian surgery, congenital uterine anomaly, intrauterine adhesion and male partner with non-obstructive azoospermia.

Beginning in February 2017, a corifollitropin alfa/PPOS protocol (study group, corifollitropin alfa/PPOS group) was started. Before January 2017, a corifollitropin alfa/GnRHant protocol (control group, corifollitropin alfa/GnRHant group) was routinely used. From August 2015 to January 2017, a total of 160 patients were recruited as the control group; from February 2017 to July 2018, a total of 173 patients were recruited as the study group.

### Ovarian stimulation in the corifollitropin alfa/GnRHant protocol

In the corifollitropin alfa/GnRHant protocol, patients received oral pills (Diane; Bayer Weimar GmbH, Weimar, Germany) in the previous cycle to induce menstruation^17^. A single dose of corifollitropin alfa 100 μg, was administered on induced menstrual cycle day 2 or day 3 (defined as stimulation day 1, S1) in the afternoon (16:00–18:00). From stimulation day 8 (S8) onward, ovarian stimulation was continued with daily rFSH injections and the dose was adjusted according to ovarian response until the day of ovulation trigger. Ovarian response was monitored using folliculometry, and serum estradiol (E2), LH, progesterone (P4) measurement. Serial serum E2, LH and P4 levels were measured on the morning of S1, S5, S8, and the day of ovulation trigger. Between S9 and the day of ovulation trigger, folliculometry and hormonal measurement were performed every 1–3 days according to ovarian response.

To prevent a premature LH surge, 0.25 mg cetrorelix (cetrotide; Merck Serono, Halle, Germany) was administered subcutaneously once daily from S5 (16:00–18:00) until the day before ovulation trigger. When more than 3 follicles had reached 18 mm in diameter, 1 mg leuprolide acetate (Lupro; Nang Kuang, Tainan, Taiwan) was administered subcutaneously to trigger final oocyte maturation, and oocyte retrieval was performed 36 h later. Vitrification of good blastocysts was performed on day 5 or day 6 after oocyte retrieval^18^. Our working routine including ultrasound and hormonal monitoring, oocyte retrieval and embryo transfer was 6 days per week.

### Ovarian stimulation in the corifollitropin alfa/PPOS protocol

Dydrogesterone replaced cetrorelix for the prevention of premature LH surge in the corifollitropin alfa/PPOS protocol. Dydrogesterone 10 mg two times per day was prescribed from S1 until the day of ovulation trigger. Other clinical management including gonadotropins stimulation and dosage tailoring, monitoring schedule of ovarian response, criteria for ovulation trigger, GnRHa triggering final oocyte maturation and oocyte retrieval was the same as that in the corifollitropin alfa/GnRHant protocol.

### Follow-up after oocyte retrieval

Patients were assessed for signs and symptoms of OHSS at 3 and 6 days after oocyte retrieval according to history taking, physical examination, and ultrasound scan. The severity of OHSS was assessed according to the classification described by Golan et al.^19^. If moderate clinical features of OHSS were present, complete blood count, coagulation profile, serum creatinine, serum electrolyte and liver function were assessed. follow up was performed every 48 h to assess the progression of moderate OHSS.

### Embryo vitrification/thawing and replacement

All blastocysts were vitrified on day 5 or 6. Good blastocysts were defined as blastocyst expansion and hatching status 3–6, inner cell mass and trophectoderm grade A or B^20^. The vitrification/thawing protocol was cryotop method (Kitayazo, Japan) based on the method described by Kuwayama^21^. FET was performed in the subsequent cycle, as described previously^18^.

### Outcome variable

The primary outcome measure was the duration of reduction in GnRHant injections. Secondary outcome measures included the incidence of premature LH surge, incidence of OHSS, additional duration/dose of daily gonadotropin administration, number of oocytes retrieved, fertilization rate, number of embryos frozen, implantation rate, clinical pregnancy rate, and live birth rate in the first FET cycle. The incidence of premature LH surge was defined as a serum LH level of ≥ 10 IU/L and P4 level of ≥ 1.0 ng/L before reaching the ovulation trigger criteria^22^. Implantation rate was calculated as the number of fetal cardiac activity detected via transvaginal ultrasound at 7 weeks of gestation divided by the number of embryos transferred. Clinical pregnancy was defined as the presence of fetal cardiac activity by transvaginal ultrasound at 7 weeks of gestation. Live birth was defined as delivery of a live child after 24 weeks of gestation.

### Statistical analysis

Statistical analysis was performed using Statistical Package for the Social Sciences (Release 10.0; SPSS). Continuous variables are expressed as mean and standard deviation (SD). The independent-sample *t* test or paired *t* test was used for continuous variables as appropriate. Categorical variables, are expressed as raw frequencies with corresponding percentages, and the between-group differences were assessed using either the chi-square test with Yates correction if required, or the Fisher exact test. A P value less 0.05 was considered to be statistically significant.

### Ethical approval

The study was approved by the Ethics Committee of National Taiwan University Hospital (201811078RINB). All patients who entered the IVF/ICSI cycles at the beginning had signed the informed consent. All methods were performed in accordance with the relevant guidelines and regulations of the Institution.

## Results

A total of 333 cycles met the inclusion criteria for analysis: 173 in the study group (corifollitropin alfa/PPOS group); and 160 in the control group (corifollitropin alfa/GnRHant group). All patients underwent their first IVF/ICSI cycles. No significant differences in terms of demographic data and baseline characteristics, including age, body weight, body mass index (BMI), proportion of primary infertility, duration of infertility, anti-Mullerian hormone (AMH) and baseline hormonal levels were observed between the two groups (Table 1).

**Table 1:** Patient demographics and baseline characteristics.

Characteristics	Corifollitropin alfa/PPOS protocol (n = 173)	Corifollitropin alfa/GnRHant protocol (n = 160)	*P* value
Total no. of cycles	173	160	
Age (y)	34.2 ± 2.8	34.4 ± 2.6	0.549
Body weight (kg)	55.8 ± 3.8	55.7 ± 3.7	0.795
Body mass index (kg/m^2^)	22.4 ± 1.8	22.4 ± 1.7	0.950
Duration of infertility (y)	3.4 ± 1.5	3.5 ± 1.6	0.844
Proportion of primary infertility (%)	80.1 (140/173)	85.0 (136/160)	0.382
AMH (ng/mL)	9.7 ± 4.2	10.1 ± 5.3	0.401
**Baseline hormonal profiles**			
FSH (IU/L)	5.7 ± 1.4	5.8 ± 1.5	0.286
LH (IU/L)	6.5 ± 2.3	6.1 ± 2.5	0.166
E2 (pg/mL)	40.2 ± 17.6	41.8 ± 16.8	0.392
Progesterone (ng/mL)	0.65 ± 0.25	0.69 ± 0.22	0.149
Testosterone (ng/dL)	34.5 ± 18.0	35.6 ± 17.4	0.553

Values are expressed as number, mean ± standard deviation or percentage.

*PPOS* progestin primed ovarian stimulation, *GnRHant* GnRH antagonist, *AMH* anti-Mullerian hormone, *FSH* follicle-stimulating hormone, *LH* luteinizing hormone, *E2* Estradiol.

**Figure 1:**
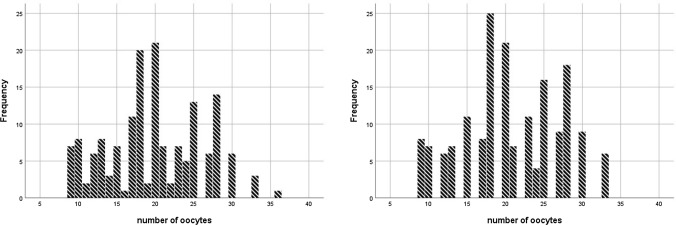
Frequency distribution of number of oocytes retrieved in the two protocols.

As shown in Table 2, no patients in either group experienced premature LH surge and OHSS. There was a significantly longer duration of GnRHant injections in the corifollitropin alfa/GnRHant group compared with the corifollitropin alfa/PPOS group. An average of 3.9 and 3.8 days of additional daily rFSH injections were required in the corifollitropin alfa/PPOS group and corifollitropin alfa/GnRHant group, respectively (P > 0.05). The additional total and average daily amount of rFSH consumption were also similar in both groups. No patients in either group experienced premature LH surge and OHSS. Other clinical outcomes with regards to serum hormonal levels on ovulation trigger day, numbers of oocyte retrieved, rate of metaphase II (MII) oocytes, fertilization rate, number of blastocyst frozen and number of good blastocysts are summarized in Table 2. Figure 1 showed the frequency distribution of number of oocytes retrieved in the two groups of patients. The clinical outcomes of the first FET cycle are presented on Table 3. There were 14 (14/173 [8.1%]) and 12 (12/160 [7.5%]) patients with no blastocyst available to be frozen in the corifollitropin alfa/PPOS group and corifollitropin alfa/GnRHant group, respectively. All patients who had embryos frozen proceeded to their first FET cycle. The clinical pregnancy (62.4% vs 60.6%), implantation (55.8% vs 52.5%), ongoing pregnancy (51.4% vs 50.0%), and live birth (49.1% vs 48.1%) rates in the first FET were similar between the two groups of patients.

**Table 2:** Characteristics of ovarian stimulation, oocyte retrieval, fertilization, embryo development and embryo freezing.

Characteristics	Corifollitropin alfa/PPOS protocol (n = 173)	Corifollitropin alfa/GnRHant protocol (n = 160)	*P* value
**Stimulation**			
Duration of GnRHant injections (days)	0 ± 0	6.8 ± 1.4	0.000
Duration of additional gonadotropin stimulation (days)	3.9 ± 1.3	3.8 ± 1.4	0.240
Total dose of additional gonadotropin consumption (IU)	497.7 ± 233.1	478.4 ± 301.8	0.511
Average dose of additional daily gonadotropin consumption (IU)	124.3 ± 32.6	120.7 ± 36.9	0.344
**Serum hormonal level on ovulation trigger day**			
E2 (pg/mL)	7694.5 ± 3219.5	7829.9 ± 3266.1	0.704
LH (IU/L)	1.5 ± 1.2	1.3 ± 1.0	0.242
Progesterone (ng/mL)	2.1 ± 0.9	2.0 ± 0.9	0.589
Incidence of premature LH surge (%)	0 (0/173)	0 (0/160)	
Incidence of OHSS (%)	0 (0/173)	0 (0/160)	
**Oocyte retrieval**			
No. of oocytes retrieved	20.7 ± 6.3	19.8 ± 6.2	0.195
Metaphase II oocyte rate (%)	78.8 ± 15.0	78.1 ± 15.8	0.688
Fertilization rate (%)	73.9 ± 16.7	73.6 ± 17.7	0.883
**Embryo development (day 5/6)**			
No. of good blastocyst	4.1 ± 3.0	4.0 ± 3.1	0.673
No. of blastocyst frozen	6.9 ± 4.8	6.7 ± 4.8	0.655

Values are expressed as either mean ± standard deviation or percentage. *PPOS* progestin primed ovarian stimulation, *GnRHant* GnRH antagonist, *E2* estradiol, *LH* luteinizing hormone, *OHSS* ovarian hyperstimulation syndrome.

**Table 3:** Clinical outcomes of first frozen embryos transfer cycle.

Characteristics	Corifollitropin alfa/PPOS protocol (n = 173)	Corifollitropin alfa/GnRHant protocol (n = 160)	*P* value
No. of started cycles	173	160	
Percentage of patients without frozen embryos (%)	8.1 (14/173)	7.5 (12/160)	1.0
No. of thawing cycles	159	148	
No. of replacement cycles	159	148	
No. of embryos transferred	1.4 ± 0.5	1.3 ± 0.5	0.792
Clinical pregnancy rate per started cycle (%)	62.4 (108/173)	60.6 (97/160)	0.737
Ongoing pregnancy rate per started cycle (%)	51.4 (89/173)	50.0 (80/160)	0.827
Live birth rate per started cycle (%)	49.1 (85/173)	48.1 (77/160)	0.913
No. of singleton	72	64	
No. of twins	13	13	

Values are expressed as number, mean ± standard deviation or percentage. *PPOS* progestin primed ovarian stimulation, *GnRHant* GnRH antagonist.

**Figure 2:**
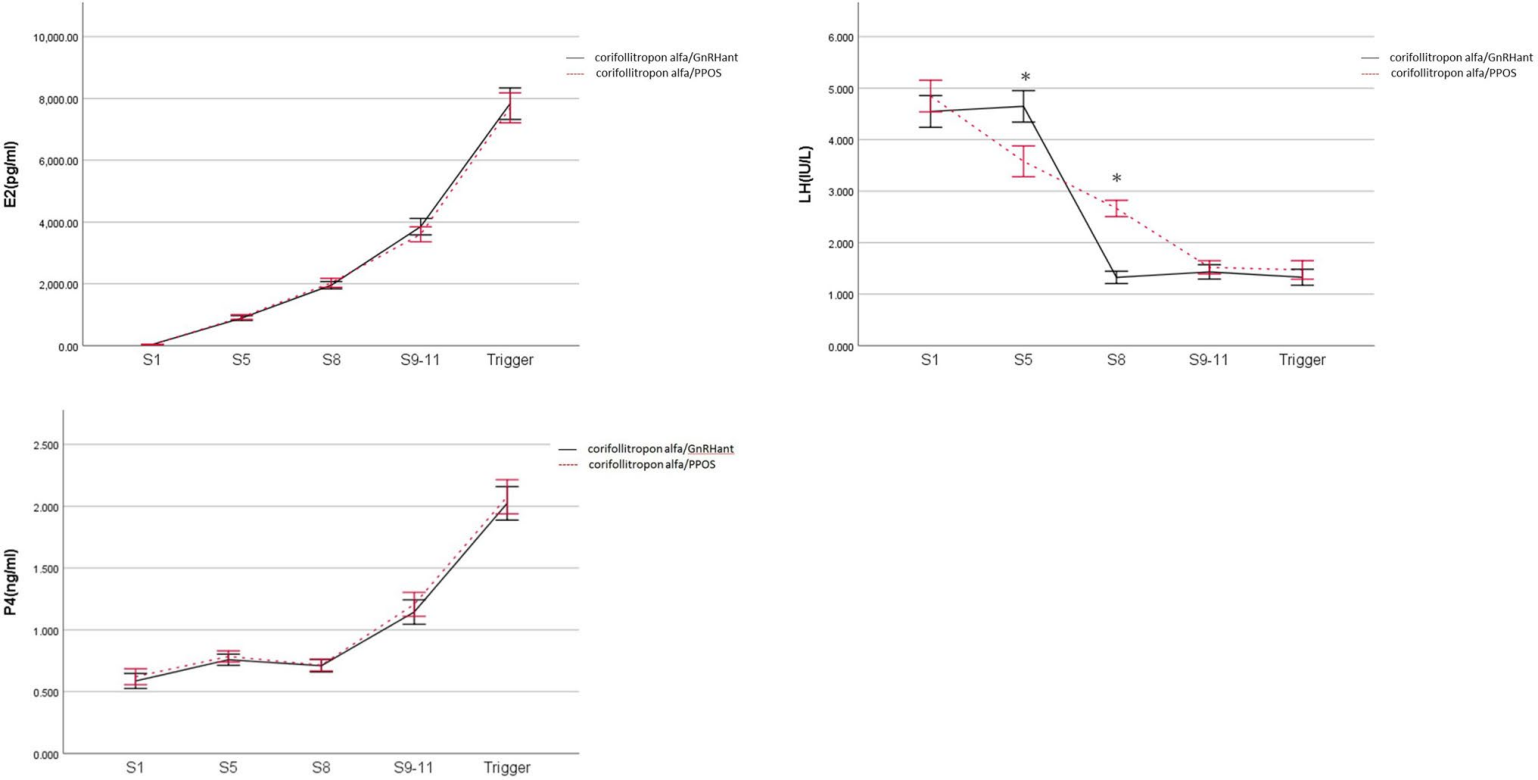
Serum hormone profiles during controlled ovarian stimulation in the two protocols. The solid black lines represent the corifollitropin alfa/GnRHant protocol, and the dashed red lines represent the corifollitropin alfa/PPOS protocol. The values were expressed as mean and standard deviation. The asterisk (*) represents *P* < 0.05 at time point. *S1* stimulation day 1, *Trigger* ovulation trigger day.

Hormonal profiles of the two groups during COS are presented in Fig. 2. In the corifollitropin alfa/PPOS group, dydrogesterone administration began from S1. Serum LH level decreased gradually from S1 to S9–11, then stabilized till the day of ovulation trigger. The corifollitropin alfa/PPOS group had a significantly lower serum LH level on S5 compared with the corifollitropin alfa/GnRHant group, whose GnRHant had not begun in the morning of S5. In corifollitropin alfa/GnRHant group, the first dose of GnRHant was administered in the afternoon of S5 and the serum LH level on S8 was significantly decreased as compared to the serum LH levels on S1 or S5. On S8, GnRHant also resulted in a significantly lower serum LH level in corifollitropin alfa/GnRHant group compared with the corifollitropin alfa/PPOS group. However, the serum LH level became similar between the two protocols on S9–11 and the day of ovulation trigger. There were no significant difference of the serum E2 and P4 profiles during COS between the two groups.

## Discussion

This study demonstrated that the corifollitropin alfa/PPOS protocol could be a simplified, patient friendly regimen for women with PCOS undergoing IVF/ICSI treatment. No patients experienced premature LH surge. The injection burden of GnRHant and gonadotropins was decreased. The risk for OHSS was minimized and the clinical outcomes appeared to be satisfactory.

The COS during IVF/ICSI treatment typically requires daily injection of gonadotropin to recruit more oocytes. This causes physical burden and psychological stress in patients, and also increases the risk for injection errors^23^. A dropout rate of > 50% has been reported by several studies even though the treatment costs could be reimbursed^24–26^. Schroder et al. reported a dropout rate of 39.9% after failure of the first cycle, which then increased to 62.2% after the fourth cycle, indicating the high level of distress and frustration experienced by patients during the IVF/ICSI treatment^24^. Substantial psychological stress and physical burden associated with conventional ovarian stimulation are the major causes for dropout before achieving pregnancy, which in turn reduces cumulative pregnancy rates^24,27–29^. Moreover, PCOS women experience high levels of emotional stress^30^, and their IVF cycles have the characteristics of longer period of COS and higher cancellation rate due to impending OHSS or insufficient ovarian response^31^. Therefore, it is crucial to develop a simple treatment regimen that mitigates the physical burden and psychological stress in women with PCOS^13^. Corifollitropin alfa is a feasible option to reduce the injection burden of gonadotropins.

Corifollitropin alfa was introduced into clinical practice before the popularity of the GnRHa trigger/freeze-all strategy and, hence, was not recommended to be used in women with PCOS in whom an excessive ovarian response was a concern. Currently, it is well recognized that the GnRHa trigger/freeze-all strategy substantially eliminates OHSS in high responders^32,33^. Thus, long-acting FSH is a reasonable alternative to conventional daily gonadotropin injections for women with PCOS because the risk for OHSS can be minimized. Corifollitropin alfa combined with a GnRHa trigger/freeze-all strategy has seldom been studied in women with PCOS or other high responders. We described a corifollitropin alfa/GnRHant protocol in PCOS patients undergoing IVF/ICSI treatment with satisfactory clinical outcomes and low risk for OHSS. Nevertheless, an average of 6.7 days of GnRHant injections was still needed to prevent premature LH surge^13^.

La Marca and Capuzzo reported that PPOS is shown to effectively prevented premature LH surge, with similar clinical outcomes compared with conventional ovarian stimulation^15^. A PPOS approach is especially suitable for high responders because a freeze-all strategy is mandatory^15^. Several oral progestins have been reported to be successfully used in women with PCOS using a PPOS protocol, including MPA^34–36^, MIP^37^, and dydrogesterone^38–40^. Daily injections of human menopausal gonadotropin (hMG) or rFSH, rather than corifollitropin alfa, was used in these studies. Our study demonstrated that co-administration of dydrogesterone with corifollitropin alfa could also effectively prevent premature LH surge in women with PCOS to further reduce the injection burden of GnRHant, meanwhile obtaining comparable clinical outcomes to those achieved using a corifollitropin alfa/GnRHant protocol. The number of additional injections needed for this protocol during COS were an average of 3.9 days of daily gonadotropin and 1 dose of GnRHa to trigger ovulation. There were no studies comparing early and late start of dydrogesterone on IVF outcomes. According to Kuang’s study, if MPA was started during the midfollicular phase in patients with multiple growing follicles and elevated serum E2 levels, the blockade of LH surge could fail^14^. In our study, we started dydrogeterone on stimulation day 1 for fear of failing to prevent a premature LH surge.

Two previous studies compared dydrogesterone primed ovarian stimulation with GnRHant protocols for women with PCOS undergoing IVF/ICSI treatment^38,40^. Eftekhar et al. conducted an RCT that recruited 60 patients receiving dydrogesterone primed ovarian stimulation and 60 patients receiving the GnRHant protocol. The number of MII oocytes, fertilized oocytes and the trigger day serum E2 levels were significantly lower in the PPOS group than in GnRHant group. The trigger day serum LH levels was significantly higher in the PPOS group^38^. Gurbuz et al. retrospectively analyzed 525 patients, of whom 258 were treated using a dydrogesterone primed ovarian stimulation protocol and 267 treated using a GnRHant protocol^40^. Similar clinical outcomes, including the incidence of premature LH surge, were reported, except for a significantly higher serum LH levels on the ovulation trigger day in the PPOS group. Although the two studies reported that dydrogesterone had a lower pituitary suppression effect compared to GnRHant, we found that both medications resulted in similar serum LH levels on the ovulation trigger day. In our study, as shown in Fig. 2, co-administration of dydrogesterone with corifollitropin alfa resulted in a steadily decrease in serum LH levels from S1 to the trigger day, while a rapidly decrease in LH levels was observed one day after GnRHant administration in the GnRHant group. On the trigger day, both regimens resulted in similar LH levels. In short, all of these studies suggest that dydrogesterone is an effective oral medication for the prevention of premature LH surge in women with PCOS undergoing IVF/ICSI treatment.

The starting dose of gonadotropin in women with PCOS undergoing IVF/ICSI treatment has not been extensively studied. To our knowledge, there have been no RCTs investigating the optimal starting doses of gonadotropins for patients at high risk for developing OHSS^41^. Thakre and Homberg proposed that gonadotropin doses should be lower than conventional doses (no greater than 150 IU), at least in the first cycle, to lower the risk for excessive response^9^. We used corifollitropin alfa 100 μg in this study because the potency was equivalent to 150 IU rFSH according to the ENSURE trial^42^. In the present study, an average of 20.7 oocytes were retrieved, and no patients experienced OHSS or other major complications, such as internal bleeding or ovarian torsion. It indicated that corifollitropin alfa 100 μg was suitable for this group of women with PCOS. In a systematic review addressing the topic of COS for freeze-all cycles, Mizrachi et al. concluded that there was strong evidence showing that the cumulative live birth rate increased with the number of oocytes retrieved, and a goal for the recovery of 15–20 oocytes in freeze-all cycles would be an acceptable balance between safety and efficacy^43^. Patients with a body weight < 50 kg or > 70 kg were excluded from the present study because we usually prescribed 112.5 IU and 200 IU rFSH, respectively, to these patients during the first 4 days of COS, then adjusted according to ovarian response. Further studies exploring the optimal corifollitropin alfa dose for these patients, therefore, are warranted.

Dydrogesterone was chosen to prevent premature LH surge in this study because it has been used for decades in pregnant women who experienced recurrent pregnancy loss and threatened miscarriage^44^. Besides, a recent systematic review and individual participant data meta-analysis comparing dydrogesterone and vaginal progesterone for IVF luteal phase support showed similar safety parameters for the mother and the fetus between the two treatments^45^. Recently, a retrospective cohort study reported similar neonatal outcomes and incidence of major congenital malformation in 1429 live-born infants after dydrogesterone primed ovarian stimulation, compared with 2127 live-born infants after a GnRHa short protocol for IVF^46^. Compared with MIP, dydrogesterone will not elevate the serum progesterone levels and interfere with the interpretation of premature luteinization during COS^47^.

There were four studies comparing IVF outcome data among different progestin formulations undergoing PPOS protocol^39,47–49^. Huang et al. retrospectively compared MPA 10 mg/day and dydrogesterone 20 mg/day in women with PCOS^39^, while Yu et al. included women with normal ovulatory women in their RCT^47^. Both studies showed similar ovarian response and clinical outcomes. However, higher gonadotropin consumption was observed in MPA than in dydrogesterone which might be due to stronger pituitary suppression by MPA^50^. Zhu et al. conducted a RCT comparing dydrogesterone 20 mg/day with MIP 100 mg/day in normal ovulatory women^48^. Guo et al. reported a retrospective study comparing MPA 10 mg/day with MIP 200 mg/day in normal ovulatory women^49^. Both the studies showed similar clinical outcomes in terms of different progestin^48,49^. In a recent systemic review including meta-analysis, similar clinical outcomes including effectiveness of preventing premature LH surge in PPOS protocol using MPA, MIP and dydrogesterone were reported^50^.

The major drawback to our study was its retrospective design. However, no changes in staff, laboratory protocols, and clinical practice occurred during the study period. All patients received their first IVF/ICSI treatment cycle. The results of this study may have some implications for daily clinical practice and further research. This protocol also could be applied to non-PCOS high responders, in whom a freeze-all strategy has been reported to result in significantly higher live birth rates and lower risk for OHSS as compared to fresh embryo transfer^51,52^.

In summary, results of the present study demonstrated that a corifollitropin alfa/PPOS protocol could effectively prevent premature LH surge while reducing the injection burden of GnRHant and gonadotropins. It has the potential to be a simplified, patient friendly protocol for women with PCOS undergoing IVF/ICSI treatment. The clinical outcomes appeared to be promising. Further RCTs are needed to compare clinical outcomes, including OHSS prevention and cumulative live birth rate with the conventional GnRHant protocol using daily administration of gonadotropins. 

## Supplementary Information

Supplementary Information.
