# Supplementary material for: Progestin primed ovarian stimulation using corifollitropin alfa in PCOS women effectively prevents LH surge and reduces injection burden compared to GnRH antagonist protocol
Source: Sci Rep. 2021 Nov 23;11:22732. doi: 10.1038/s41598-021-02227-w (PMC8611037; doi:10.1038/s41598-021-02227-w)
Supplement: Supplementary file 1 — Supplementary Information. [file 41598_2021_2227_MOESM1_ESM.docx]

Figure 1. Study flow diagram.

142 PCOS patients whose body weight were not between 50~70 kg were excluded

400 non-first IVF/ICSI cycle PCOS patients were ecxcluded

5,809 non- PCOS patients were excluded from initial survey

875 PCOS patients were screened

333 PCOS patients weighing between 50~70 kg who underwent their first IVF/ICSI cycle were recruited for final analysis

475 PCOS patients were of their first IVF/ICSI cycle and were assessed for eligibility

Total 6,684 IVF /ICSI cycles were conducted between August 2015 to July 2018
